# GIRK channels in Alzheimer’s disease

**DOI:** 10.18632/aging.104026

**Published:** 2020-10-14

**Authors:** Ori J. Lieberman, Francesca Bartolini, Maria Concetta Miniaci

**Affiliations:** 1 Department of Psychiatry, Columbia University Medical Center, New York, NY 10032, USA; 2 Medical Scientist Training Program, Columbia University Vagelos College of Physicians and Surgeons, New York, NY 10032, USA; 3 Department of Pathology & Cell Biology, Columbia University Medical Center, New York, NY 10032, USA; 4 Department of Pharmacy, University of Naples Federico II, Naples, Italy

**Keywords:** GIRK channels, Alzheimer’s disease, neuromodulators

G protein-coupled inwardly rectifying K+ (GIRK) channels are a family of ion channels that are activated by GPCRs responsive to dopamine, serotonin, opioids, and γ-aminobutyric acid (GABA) [1]. Upon activation, GIRK channels mediate an outward potassium current that hyperpolarizes neuronal membranes and decreases neuronal intrinsic excitability. Due to their rectifying nature, GIRK currents facilitate membrane bistability as their outward potassium current is shut down following sufficient excitation or changes in neuromodulatory tone, yielding synaptic circuits that are “trained” to be in one of two states: either silent even in response to moderate drive, or very active in response to sufficient excitation. This bistable state is hypothesized to establish the ability to learn new tasks during juvenile periods and access and retain them during maturity.

One critical aspect of Alzheimer’s disease (AD) is the reorganization of brain activity that determines network hyperexcitability, epileptiform spikes, and loss of synaptic plasticity at the early stages of disease [2]. Excitatory glutamatergic neurotransmission via N-methyl-d-aspartate receptor (NMDAR) is essential for synaptic plasticity and neuronal viability. Excessive NMDAR activity, however, causes excitotoxicity and cell death, underlying a potential cellular mechanism of neurodegeneration and suggesting that loss of “bistable” membrane potential may contribute to neuronal dysfunction in AD.

Pathophysiological alterations of GIRK channels has been demonstrated in AD. *In vitro* and *in vivo* studies indicate that amyloid beta1-42 (Aβ), the peptide that accumulates in AD brains in its insoluble form [3], alters the expression and activity of GIRK channels while the selective pharmacological activation of GIRK channels rescues synaptic plasticity and memory deficits in animals injected with Aβ [4]. Most of these studies, however, have evaluated the effect of Aβ in an acute animal model of neurodegeneration thus leaving many open questions. For example, whether and how GIRK channel dysfunction contribute to the dysregulation of firing homeostasis and impairment of synaptic plasticity seen at various stages of AD remains unexplored. Evaluation of GIRK impact on network dysfunction in AD should take into consideration two important aspects: cell-type-specific differences in GIRK channel resting conductance and the potential decline in control of GIRK currents by neuromodulators over the disease course. For example, dorsal hippocampal neurons have a higher resting GIRK conductance and, therefore, a higher threshold for long-term potentiation (LTP) compared to ventral neurons, which can undergo potentiation even in response to weak and temporally dispersed excitatory inputs [5]. In the cerebellum, we have recently demonstrated that GIRK channels have a marked effect on synaptic plasticity since the application of GIRK channel agonist ML297 increased the expression of LTP while preventing long-term depression [1]. Based on these observations, it would be interesting to understand how Aβ affects GIRK channels in different brain areas in the transgenic (Tg) mouse model of AD at different stages of the disease.

Another important feature of GIRK channels is that they represent a site of convergence for different neuromodulators permitting continuous adaptation to the current task and promoting the refinement of the neural network required for memory storage and higher cognitive functions. Since AD is associated with robust decreases in cortical cholinergic innervation, as well as changes in other neuromodulators including norepinephrine [6], it would be interesting to examine the potential relationship between changes in the levels of neuromodulators, alterations of GIRK currents, and variations in neuronal activity. Indeed, we have recently demonstrated that the early depletion of norepinephrine in the cerebellum of Tg AD mice is associated with synaptic plasticity impairment at the parallel fiber-Purkinje cell synapse as well as motor coordination and balance deficits in prodromal AD [7,8]. Defining the intersection between neuromodulatory state and GIRK function in AD will shed light on the mechanism through which drugs that affect neuromodulation may have a benefit in AD while paving the way to the development of novel therapeutic approaches aimed at ameliorating synaptic dysfunctions and cognitive decline in this disease.
